# Cold Water Immersion After a Handball Training Session: The Relationship Between Physical Data and Sensorial Experience

**DOI:** 10.3389/fspor.2020.581705

**Published:** 2020-10-20

**Authors:** Maxime L'Hermette, Ingrid Castres, Jeremy Coquart, Montassar Tabben, Nihel Ghoul, Bernard Andrieu, Claire Tourny

**Affiliations:** ^1^University of Rouen UFR STAPS, Cetaps EA, Rouen, France; ^2^Aspetar Orthopaedic and Sports Medicine Hospital, Doha, Qatar; ^3^University of Paris Descartes, Paris, France

**Keywords:** athletic recovery, handball, physiological data, sensations, cold water immersion, verbatim reports, experiential data

## Abstract

The aim of this study was to examine the relationship between the physiological data from subjects and their reported sensory experiences during two types of recovery methods following a handball training session. Female handball players (average age: 21.4 ± 1.3 years; weight: 59.2 ± 3.3 kg; height: 158 ± 3 cm; body mass index, 23.4 ± 2.0 kg.m^−2^) carried out an athletic training session (rating of perceived exertion RPE: 14.70 ± 0.89) with either a passive recovery (PR) period or cold water immersion (CWI) for 14 min) (cross-over design). Physiological data were collected during the recovery period: CWI had a greater effect than PR on heart rate (HR; bpm), the higher frequencies (HF) of heart rate variability (HRV: 46.44 ± 21.50 vs. 24.12 ± 17.62), delayed onset muscle soreness (DOMS: 1.37 ± 0.51 vs. 2.12 ± 1.25), and various reported emotional sensations. Spectrum HRV analysis showed a significant increase in HF during CWI. Sensorial experiences during the recovery periods were gathered from verbatim reports 24 h later. Players' comments about CWI revealed a congruence between the physiological data and sensorial reports. They used words such as: “*thermal shock*,” “*regeneration*,” “*resourcefulness*,” “*dynamism*,” and “*disappearance of pain*” to describe their sensations. In conclusion, this study demonstrated the link between physiological and experiential data during CWI and we propose that action of the parasympathetic system on the autonomic nervous system can, at least in part, explain the observed correlations between the corporeal data measured and the sensorial experiences reported.

## Introduction

Athletes often experience high levels of fatigue due to high training-loads, as well as frequent competitions (Hausswirth et al., 2011; Versey et al., 2013). Fatigue is multi-dimensional and related to factors such as psychological tension or mild inflammatory disorders (Versey et al., 2013). For the purposes of this study, fatigue is defined as a feeling of exhaustion with a decline in physical performance (Montgomery et al., 2008).

One method to limit the effects of fatigue on performance is cold water immersion (CWI). CWI is an established method of recovery for athletes who carry out intermittent sports (Montgomery et al., 2008; Rowsell et al., 2009; Ascensao et al., 2011).

We wished to investigate the relationship between the corporeal (i.e., physiological data) and sensorial experiences (i.e., described from verbatim descriptions) of handball players during CWI following a training session. The sensorial data was evaluated using a method previously described by Andrieu and Gerardin (2012) called “emersiology” where “emersiology” was defined as “*a reflexive science dealing with the emersion of sentient life from the consciously experienced body*” where “*emersion is the involuntary movement, within our bodies, of connections, humors and images of which only the tip reaches our awareness*” (Andrieu and Gerardin, 2012; Andrieu and Burel, 2014).

In traditional, Western medicine, the corporeal or living body is evaluated by physiological parameters such as delayed onset muscle soreness (DOMS), the subjective intensity of the training session (rating of perceived exertion, RPE), and heart rate variability (HRV) which is the variation in the time interval between heartbeats (Haddad et al., 2013). HRV provides a non-invasive evaluation of autonomic control of the heart (Buchheit et al., 2009) and is sensitive to factors such as fatigue, physiological and psychological stress, and has also been shown to be related to respiratory activity. Respiration affects HRV, producing low frequency (LF) and high frequency (HF), respectively, above and below the breathing frequency (BF) threshold of 0.15 Hz. Fluctuations above 0.15 Hz are associated with vagal activity, whereas fluctuations between 0.04 and 0.15 Hz have been reported to be mediated by both vagal and sympathetic cardiac nerves (Perini and Veicsteinas, 2003; Saboul et al., 2014).

CWI is used to accelerate recovery so that performance can be restored to normal as soon as possible (Montgomery et al., 2008; DeMartini et al., 2011; Bastos et al., 2012; Versey et al., 2013). The effects of CWI are greater than active recovery because of the effects of hydrostatic pressure and the water temperature (Barnett, 2006; Versey et al., 2013). CWI has been shown to increase blood flow and decrease blood lactate concentrations. This reduces fatigue and enables higher training loads to be sustained (Pastene et al., 1996). The CWI protocols described in the literature describe the immersion of either the lower limbs or the whole body, and the temperatures used vary from 10 to 15°C (Montgomery et al., 2008; Buchheit et al., 2009; Ascensao et al., 2011; Bastos et al., 2012; Stanley et al., 2012; Versey et al., 2013). The time of immersion also varies from 3 to 20 min, and involves either one continuous immersion or several immersions, each of 1–5 min with 1–2 min in between (Bailey et al., 2007; Hausswirth et al., 2010; Parouty et al., 2010; Ascensao et al., 2011; Bleakley et al., 2012; Elias et al., 2013).

To our knowledge, none of these previous studies have examined the use of CWI in intermittent sports on both athletes' physical or corporeal performance and on their sensorial experiences. Thus, the purpose of this study was to analyse the rapport between the corporeal and sensorial experienced bodies after CWI. We used passive recovery (PR) data as the control and hypothesized that CWI would: (1) improve corporeal performance recovery data compared to PR after one handball training session as measured by physiological parameters and (2) receive more positive reports from subjects regarding their sensorial experiences as evaluated by the “emersiology” method.

## Materials and Methods

### Subjects

Eight healthy female handball players (age = 21.4 ± 1.3 years; body mass: 59.2 ± 3.3 kg; height: 158 ± 3 cm; body mass index: 23.4 ± 2.0 kg.m^−2^) from the French Handball First Division volunteered to take part in this study. No subjects had medical contraindications to CWI (e.g., Raynaud's syndrome or cold hypersensitivity), and all used an oral contraceptive.

All subjects signed an informed consent form before participation. The protocol was fully approved by the local scientific committee and the study was performed in accordance with the Declaration of Helsinki ethical standards.

### Procedure

**Figure 1:**
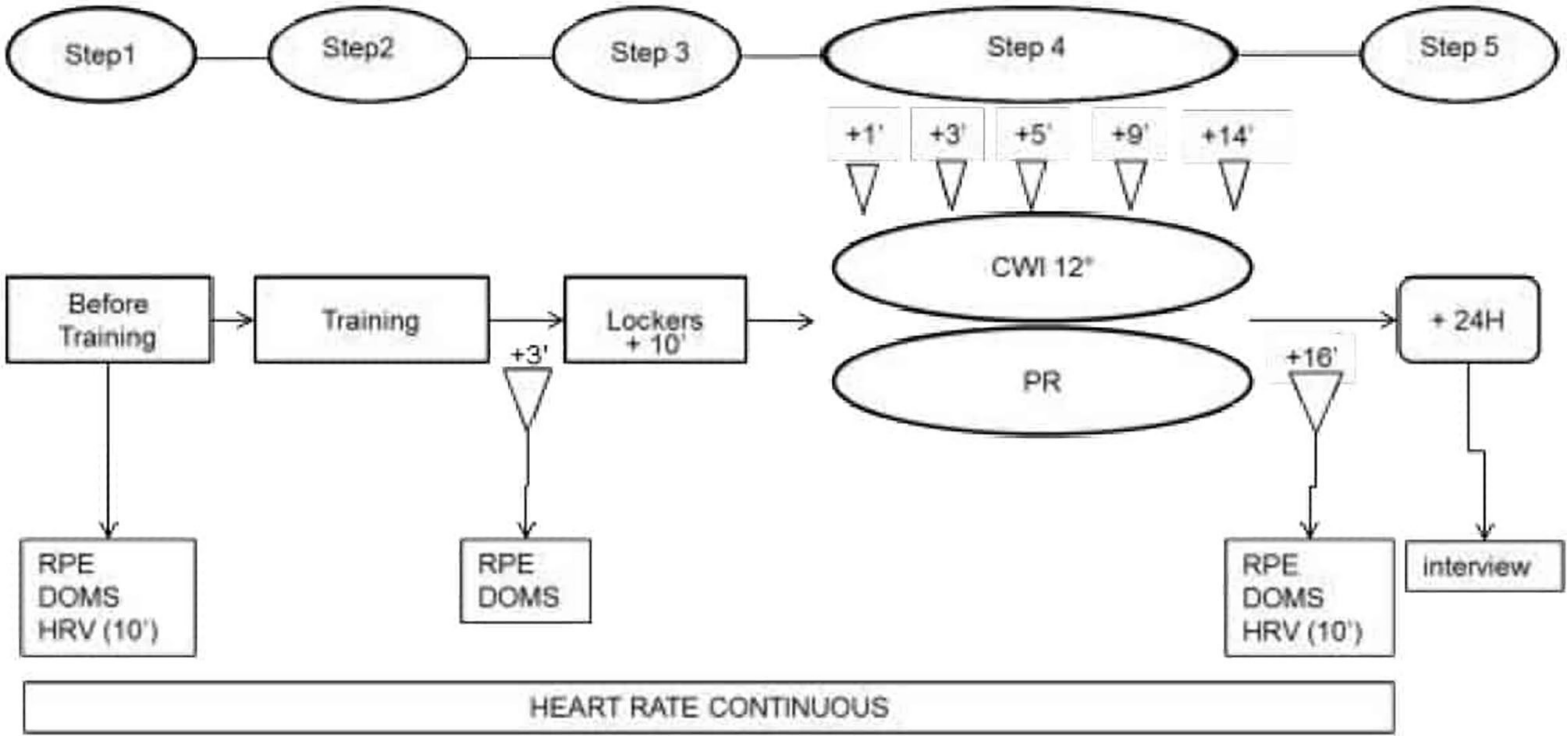
Experimental procedure. RPE, Ratings of Perceived Exertion; DOMS, Delayed Onset Muscle Soreness; HRV, Heart Rate Variability; CWI, Cold Water Immersion; PR, Passive Recovery. The interview was to gather sensorial data.

The study was carried out over 2 days (day 1 = D1; day 2 = D2) 1 week apart. The same protocol was used on both D1 and D2 and it was composed of 5 steps, as shown in Figure 1.

Subjects' heart rate was recorded throughout by a zephyr belt and HRV was recorded over 10 min with subjects lying prone on a mat on the floor with their eyes closed. DOMS was evaluated throughout the recovery periods using hooper's scale by moving a cursor along a 7-cm horizontal line and graded according to the following: 1 = no pain, 2 = very slight tiredness, 3 = slight tiredness, 4 = tender but not sore, 5 = slightly sore, 6 = sore, 7 = very sore (Haddad et al., 2013). Corporeal fatigue was evaluated by using a RPE, graded using a borg scale from 6 to 20 (Foster et al., 2001).

The training session began with a 25-min warm-up followed by 4 moderate-to-vigorous interval training exercises (20” /20”) with ball-handling or information-decision constraints: each exercise lasted 8 min and was associated with 3 min of recovery (the subjects walked at spontaneous speed around the field). The fifth and final exercise consisted of 12 min of a full handball game without any time-out (the ball was backed into play by the goalkeeper). The training session thus lasted 1 h and 21 min in total.

For the recovery, subjects were randomly divided into 2 groups (group 1 = G1; group 2 = G2). Both groups underwent both types of recovery but in opposite order, i.e., D1/G1 = PR and D1/G2 = CWI and then on D2 /G1 = CWI and D2/G2 = PR.

CWI involved plunging the subject into water up to their waist for 14 min. The water temperature was 12°C and was controlled by a digital thermometer (HI 98509; Checktemp^®^, Australia) and re-adjusted with ice if necessary. The atmospheric pressure and temperature of the room were measured using a desktop weather station (EW93, Oregon Scientific, USA).

PR involved reclining quietly on a chair for 14 min.

HR and HRV were measured at 1, 3, 5, 9, and 14 min after CWI or PR began. Sensorial data were gathered 24 h later when subjects were interviewed and asked about their perception of the recovery experience.

### HR and HRV Measurements

All subjects were equipped with a Zephyr Physiological Status Monitoring Training Ecoteam^®^ chest belt (Zephyr Performance Systems, Annapolis, Maryland, USA) throughout the procedure. The sampling rate was 1,000-Hz. Data were downloaded to the Zephyr software unit which visualized the HR trace and allowed extraction of a cardiac period (R-R interval) file in “.txt” format that was subsequently analyzed by HRV Analysis software (Kubios HRV, Biomedical Signal Analysis Group, Department of Applied Physics, University of Kuopio, Finland).

HRV was estimated by time-domain parameters (the root mean square difference of successive normal R-R intervals (RMSSD), the percentage of pairs of adjacent RR intervals that differed by >50 ms (pNN50) and frequency-domain analysis (HF, LF). For each subject, HRV was calculated from the last 5 min of their 10-min HR data, R-R intervals were extracted and beat-to-beat analysis was carried out. These parameters have classically been used to study autonomic nervous system (ANS) modulation by parasympathetic activity (Buchheit et al., 2009; Stanley et al., 2013). HRV analysis was restricted to indices of parasympathetic reactivation: the RMSSD, the percentage of successive R-R differences >50 ms (pNN50). The presence of high frequency HR has previously been shown to indicate influence of the parasympathetic branch of the ANS, since other work has demonstrated that high frequencies (0.15–0.50 Hz) are caused by vagal nerve activity which no longer occurs following administration of atropine (which blocks the effects of acetylcholine, a neurotransmitter released by the parasympathetic nervous system (PNS).

Improvements in athletic performance have been found to be closely correlated with an increase in spectrum power of the HFs in a lying position (Schmitt et al., 2013). Low frequencies, by contrast, have mostly been shown to reflect sympathetic activity. These two control systems are complimentary (Souza Neto et al., 2003). The LF/HF ratio calculated from these two spectral densities thus represents the vago-sympathetic balance of the ANS and this approach is used to distinguish the dominant spectral band. In athletes this ratio is <1 during warm-up and training conditions and is >1 during periods of high fatigue (Saboul et al., 2013). The ratio is, however, modulated by respiratory frequency, which is why HRV measurements also need to be made during rest (Saboul et al., 2014) and to facilitate this, HRV recordings were carried out in a quiet room in an attempt to minimize or avoid fluctuations in HR.

### Statistical Analysis

Normality of the data was confirmed using a Shapiro-Wilks test and a paired *t*-test was carried out for each recovery condition independently for the dependent variables: DOMS and HR (both before and after training) and HR (during recovery at 1, 3, 5, 9, and 14 min). A log transformation was applied to the HRV data from the RR interval to reduce bias from any non-uniform inter-individual value. Additionally, the differences were specified using a magnitude–based Cohen's effect size (ES) presented in Table 1. The ES was assessed using the following criteria: <0.2 = trivial, 0.2–0.6 = small, 279 >0.6–1.2 = moderate, >1.2–2.0 = large, and >2.0 very large differences (Hopkins, 2000).

**Table 1:** Comparison heart rate variabilities (HRV) and delayed onset muscle soreness (DOMS) scores in female handball players following passive recovery (PR) or cold-water immersion (CWI) treatment post-exertion.

		**CWI**	**PR**	**ES**
DOMS	DOMS +16 min	1.37 ± 0.517^***^	2.12 ± 1.25	0.77
	HF	46.44 ± 21.50^***^	24.12 ± 17.62	1.13
	LF	53.37 ± 21.61^***^	75.69 ± 17.65	1.13
HRV	LF/HF	5.76 ±4.921^**^	1.812 ± 1.701	1.07
	RMSSD	3.692 ± 0.726	3.396 ± 0.934	0.35
	pNN50	2.523 ± 1.29	2.2510 ± 1.37	0.20

*ES, Effect size*.

Levels of statistical significance are indicated where

** *p < 0.01*,

*** *p < 0.001*.

## Results

The mean HR (bpm) was measured during both training sessions and no significant differences were found (trivial or small ES). The average HR during training on day 1 was 80.3 ± 17.8% HRmax and was 77.24 ± 17.2% HRmax on day 2. A further breakdown of HR post-training and post-recovery data are shown with an index of fatigue (RPE scale) in Table 2.

**Table 2:** Fatigue as measured by rating of perceived exertion (RPE) scale of 6 to 20 and heart rate (HR) values (bpm) measured post-training (postT) and post-recovery (postR) of eight female handball players.

**Condition**	**RPE_postT_**	**RPE_postR_**	**HR_postT_**	**HR_postR_**
PR	14.5 ± 3.5 NS	9 ± 3.4 NS	165.29 ± 5.2 NS	88.71 ± 13.1 NS
CWI	13.5 ± 3.0	8.5 ± 2.7	165.71 ± 6.9	85.8 ± 9.0
ES	0.30	0.16	0.06	0.25

*NS indicates no significant difference (p > 0.05)*.

*PR, passive recovery; CWI, cold water immersion; ES, effect size*.

**Figure 2:**
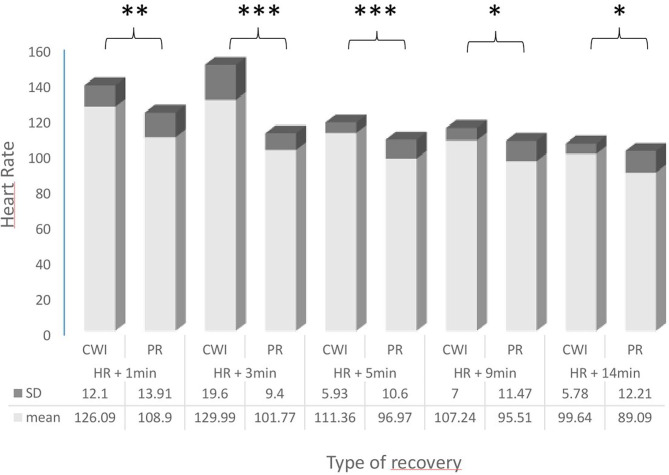
Comparison of heart rates (HR; bpm) in female handball players following passive recovery (PR) or cold-water immersion (CWI) treatment post-exertion. CWI, Cold Water Immersion; PR, Passive Recovery. Levels of statistical significance are indicated where ^*^p < 0.05, ^**^p < 0.01, ^***^p < 0.001.

The results of the parameters measured during both recovery conditions are shown in Figure 2 and Table 1, including the ES.

There was a significant difference in HR values during the recovery period; HR was significantly higher during CW1 at 1, 3, 5, 9, and 14 min compared with PR.

**Figure 3:**
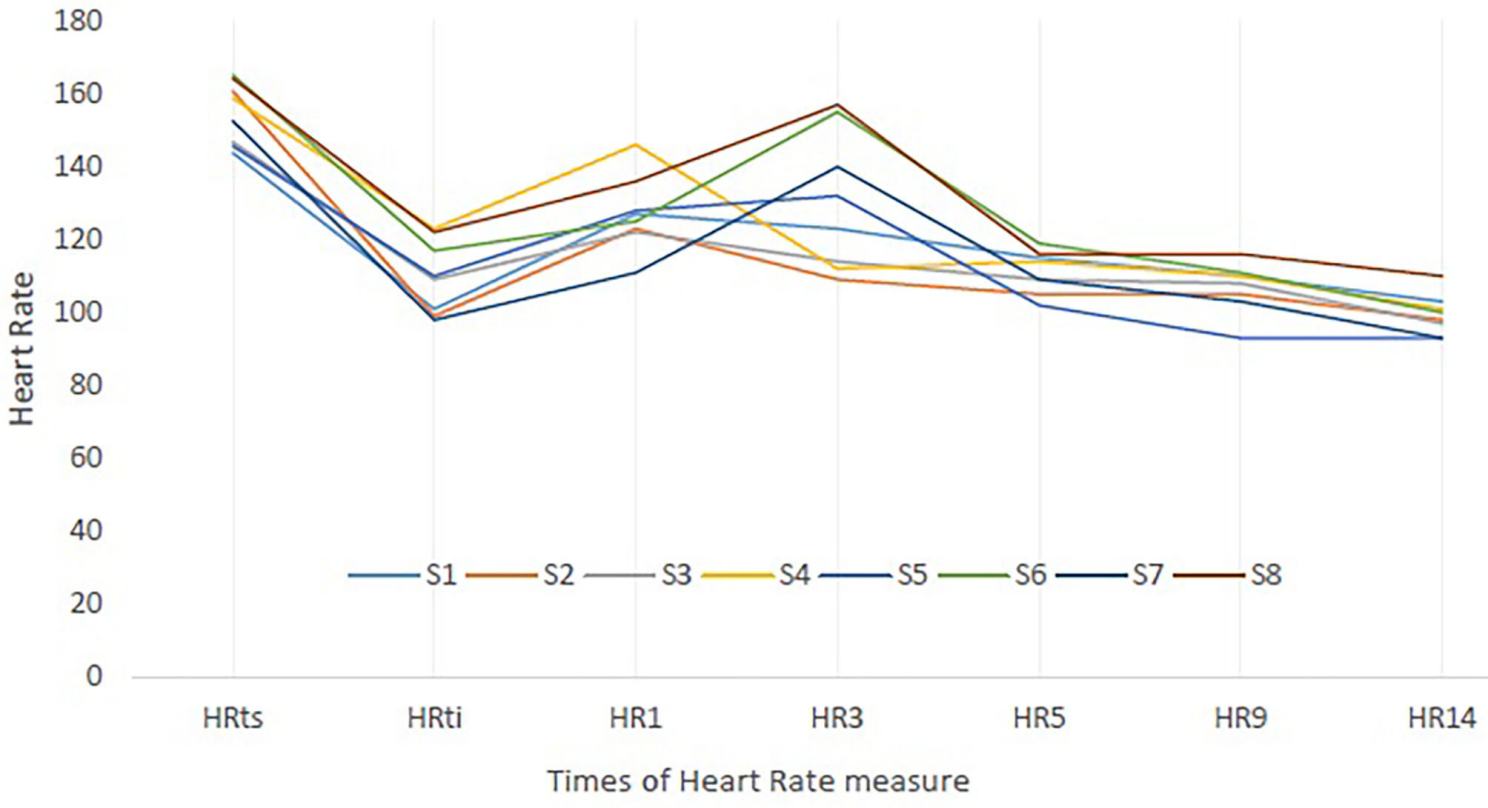
Heart rate (HR) individual's responses to training session (ts) and to cold-water immersion (CWI). S, Subjects; HRts, Mean HR of the training session; HRti, HR at initial time of immersion. HR1, HR3, HR5, HR9, HR14: HR value at 1, 3, 5, 9, and 14 min of CWI.

The HR individual's responses of the subjects to the training session (HRts) and to the CWI are presented in Figure 3. This figure highlights that the subjects have all responded to the cold shock in increasing their HR (maximal value is reached at HR1 or HR3).

At the end of the recovery periods, DOMS was significantly lower after CWI than after PR (moderate ES). Spectrum analysis of HRV showed that the number of HFs was significantly increased during CWI than during PR (*p* < 0.001; large ES), a change that was also associated with a decrease in LF (*p* < 0.001; large ES). The LF/HF ratio was thus significantly greater after CWI than after PR (moderate ES). No significant differences were found in variables measured by frequency analysis (RMSSD, pNN50) between CWI and PR (small ES).

## Discussion

This study compared the effects of CWI and PR as recovery methods for athletes following a period of intense intermittent exercise (a handball training session and match). No difference was found in HR data at the end of both types of recovery, nor in levels of reported fatigue (as measured by RPE score). The absence of differences in these two variables is important because if either parameter had significantly decreased in the period between training and recovery, it would have been impossible to ascribe any benefits reported to either type of recovery procedure with any certainty.

HRV has been shown to be a sensitive measure of cardiac activity adaptation by the ANS and is evaluated by both frequency and spectrum parameters. We found no difference in the frequency parameters of the HRV as identified by the RMMSD and pNN50 between the two recovery conditions.

With regards to the spectral HRV parameters, however, a significant increase in HF values was found for data from the CWI recovery which was accompanied by a significant decrease in LF values. These changes corroborate the strong action of the PNS on vagal inhibition during the CWI while the changes in the LF data and the LF/HF ratio suggest a concomitant reduction in sympathetic activity (Buchheit et al., 2009). One effect of PNS stimulation is the secretion of growth hormones which are involved in repair and regeneration of muscles (Stanley et al., 2013; Devesa et al., 2016). We believe that the results of this study therefore demonstrate the effectiveness of CWI on this aspect of the recovery process.

Furthermore, studies have also shown that stress and anxiety reduce the activity of the PNS and so increase sympathetic nervous system activity (Dishman et al., 2000; Shaikh al arab et al., 2012), a fact which we believe increased the importance of the increase in the number of HRV LF after CWI (Buchheit et al., 2009). This decrease in LF could also have been related to the reported feelings of peace and well-being that were described by the subjects. We propose that CWI PNS-stimulation could have been involved in the production of these sensorial experiences of new-found energy and renewed vigor.

DOMS scores decreased significantly after CWI in comparison to the scores recorded after PR. This effect, which was followed over a 14-min recovery period, is in accordance with the statement of Dupuy et al. (2018): an explanation of the impact of CWI on DOMS is a reduction in exercice-induced inflammation and muscle damage. The level of immersion and the cold temperature of the water may reduce the formation of oedema and pain sensation (Montgomery et al., 2008; Versey et al., 2013).

HR was significantly higher throughout the 14 min of CWI, compared with 14 min of PR. This may have been due to the thermal shock- or cold shock - following immersion of the lower limbs and pelvis in the cold water, which is characterized by an inspiratory gasp, hyperventilation and increased HR (Dupuy et al., 2018). Moreover, the subjects were not familiarized to CWI which improved the physiological response (Castellani and Tipton, 2015).

The whole body (including the face) submersion in cold water and the cold shock habituation cause bradycardia which reduces the arterial hypertension caused by the sudden flow of blood from the peripheral to the core vasculature in response to the low temperature (Castellani and Tipton, 2015; Park et al., 2018). In our study, only the legs and pelvis were immersed, and so it seems likely that peripheral vasoconstriction only occurred in these lower regions. In addition to hyperventilation, we propose these factors caused the observed increase HR rather than the decrease that would have been expected with total body immersion. Indeed, respiration is known to be an important regulator of cardiac rhythm; inhalation temporarily inhibits the influence of the PNS and accelerates cardiac rhythm (Bleakley and Davison, 2010; Stanley et al., 2013).

The subjects also reported a strong emotional experience with CWI. Players described a varied range of different sensations over the 14-min of CWI from the initial discomfort and then on throughout the recovery and acclimatization which represents the common reaction after the cold shock (Castellani and Tipton, 2015; Bouzigon et al., 2018). Subject described sensorial experiences included their initial shock upon first entering the water and then how their first 3 min in the cold was associated with unprecedented and intense sensations that they felt they could not immediately control. The variability between individuals in their thermoregulatory responses during cold exposure should be attributable to anthropometric differences (Bahnert et al., 2013; Castellani and Tipton, 2015). The cold creates a sensation of pain and discomfort which is due at least in part to the muscle tension and dizziness caused by hypotension following peripheral vasoconstriction (Bouzigon et al., 2018). The intensity of these painful sensations reduced over time, producing an analgesic effect and sense of well-being post-immersion (Elias et al., 2013; Park et al., 2018). The reduction in pain was progressive and subjects reported that it took them at least 3 min to acclimatize to the CWI, and around 5 min for their feelings of well-being to set-in: “*from the fifth minute, we no longer felt the cold as strongly as when we first entered the water and we had a good time, we laughed, it was more than relaxation*.” One player described how the CWI “*became bearable after 5 minutes, it was less painful, but I was not comfortable, I felt the cold, I got used to it I think. As if it paralysed my toes. I got thermal shock first, then I got used to it*.”

Another player described how, during the period of adaptation she “…*was quite cold from the 3rd minute*, [but] *by the 9th minute, I had got used to the water, my muscles relaxed*.” Subjects described how they perceived the sensations of being in the CWI as more than just the feeling of their muscles relaxing but that benefits were felt upon leaving the CWI: “*When I started to warm-up, I felt pain in my legs, but it was less intense*.”

Subjects reported how the sensation of the CWI generated memories of past sensations, such as swimming in the cold sea which highlights the relation between the sensorial experiences and the perceived sensations (Andrieu and Gerardin, 2012; Andrieu and Burel, 2014). Once acclimatized, subjects reported that the immersion in cold water was pleasurable and more relaxing than simply lying still. The immersion seemed to bring new energy, accompanied by a sensation of body vitality, never experienced before. These statements could be explain by the fact that the subjects are not habituated to the cold shock and have a strong thermoregulation responses to cold, which is attenuated after the first time of CWI (Bailey et al., 2007; Bleakley et al., 2012).

Sensorial experiences described how heat generated by the physical load of the training session contrasted with the cold shock of the CWI. This seemed to have created a sense of euphoria. The keenly felt contrast of temperatures is the sign of internal activity generating heightened perception of the body (Andrieu and Burel, 2014). The desire to return to training or physical effort is facilitated by the sensation of well-being (Montgomery et al., 2008). One player described the tension between the unpleasant sensations that were experienced with the knowledge that the CWI was good for the physical body: “*We were in the cold water, it was not very pleasant but it is good for our bodies*” and how although the awareness of being in cold water did not disappear, “*even if the cold water disturbed me less*.”

The awareness of being in cold water did not disappear but instead produced variations in pain that increasingly bearable. Subjects reported a progressive reduction in tension between the physical and sensorial experiences of the cold and we believe it was this reduction which facilitated their acceptance of the CWI as being beneficial for their health.

We acknowledge several limitations associated with the study. Whereas, cold can inhibit the inflammatory process, the overall decrease in DOMS may not be as great after 14 min of CWI. This time could ultimately moderate CWI effectiveness at reducing pain. However, the cold shock reduced soreness more effectively than PR. Due to the nature of water immersion, it was not possible to include several conditions of immersion (durations and temperatures) to determine the optimal protocol specific to each subject. Although this is a key outcome in the recovery of an athlete, further studies should consider the dose–response effect of CWI on other markers of muscle damage, such as performance, in order to identify the best CWI recovery strategy based on different and relevant factors.

## Practical Applications

DOMS scores decreased significantly after CWI in comparison to the scores recorded after PR. This effect is important for an intermittent game such as handball, as it is played with short but intense periods of exertion. A sensation of well-being that arose from the reduction in muscle pains was also described by the players 24 h after their training session. The CWI following games may help increase perceptual recovery. Players, coaches and medical staff should be aware that the use of CWI can decrease the post-training physical and psychometric loads.

## Conclusion

CWI is an efficient mean of triggering immediate, post-exercise parasympathetic activity, and reducing the DOMS. The sensorial experiences described by the subjects demonstrated a positive view of recovery using CWI. The person's sensorial experiences can influence the effectiveness of the CWI recovery as it will indicate whether or not the individual is receptive and able to respond to that potentially uncomfortable of the cold shock.

## Data Availability Statement

The raw data supporting the conclusions of this article will be made available by the authors, without undue reservation.

## Ethics Statement

The studies involving human participants were reviewed and approved by the local commitee scientific of university of Rouen. Written informed consent to participate to this study was provided by the participant. The patients/participants provided their written informed consent to participate in this study.

## Author Contributions

ML'H, JC, IC, NG, MT, BA, and CT as conceived, designed, performed, and analyzed the research. ML'H and CT wrote the manuscript. All authors read, review, and approved the final manuscript.

## Conflict of Interest

The authors declare that the research was conducted in the absence of any commercial or financial relationships that could be construed as a potential conflict of interest.
